# Reduction of endotoxin attenuates liver fibrosis through suppression of hepatic stellate cell activation and remission of intestinal permeability in a rat non-alcoholic steatohepatitis model

**DOI:** 10.3892/mmr.2014.2995

**Published:** 2014-11-24

**Authors:** AKITOSHI DOUHARA, KEI MORIYA, HITOSHI YOSHIJI, RYUICHI NOGUCHI, TADASHI NAMISAKI, MITSUTERU KITADE, KOSUKE KAJI, YOSUKE AIHARA, NORIHISA NISHIMURA, KOSUKE TAKEDA, YASUSHI OKURA, HIDETO KAWARATANI, HIROSHI FUKUI

**Affiliations:** Third Department of Internal Medicine, Nara Medical University, Kashihara, Nara 634-8521, Japan

**Keywords:** endotoxin, toll-like receptor 4, hepatic stellate cell, liver fibrosis, intestinal permeability, non-alcoholic steatohepatitis

## Abstract

Previous clinical studies have demonstrated that endotoxin/toll-like receptor 4 (TLR4) signaling is critical in the inflammatory pathways associated with non-alcoholic steatohepatitis (NASH). In human and animal studies, NASH was associated with portal lipopolysaccharide (LPS) and the plasma LPS level was hypothesized to be associated with small intestinal bacterial overgrowth, change in composition of the microbiota and increased intestinal permeability. The aim of the present study was to investigate the roles of endogenous endotoxin and TLR4 in the pathogenesis of NASH. The effects of antibiotics were assessed *in vivo* using a choline deficiency amino acid (CDAA)-induced experimental liver fibrosis model. Antibiotics, including polymyxins and neomycins, were orally administered in drinking water. Antibiotics attenuated hepatic stellate cell (HSC) activation and liver fibrosis via TGF-β and collagen in an experimental hepatic fibrosis model. The mechanism by which antibiotics attenuated LPS-TLR4 signaling and liver fibrosis was assessed. Notably, TLR4 mRNA level in the liver was elevated in the CDAA group and the CDAA-induced increase was significantly reduced by antibiotics. However, no significant differences were observed in the intestine among all groups. Elevated mRNA levels of LPS binding protein, which was correlated with serum endotoxin levels, were recognized in the CDAA group and the CDAA-induced increase was significantly reduced by antibiotics. The intestinal permeability of the CDAA group was increased compared with the choline-supplemented amino acid group. The tight junction protein (TJP) in the intestine, determined by immunohistochemical analysis was inversely associated with intestinal permeability. Antibiotics improved the intestinal permeability and enhanced TJP expression. Inhibition of LPS-TLR4 signaling with antibiotics attenuated liver fibrosis development associated with NASH via the inhibition of HSC activation. These results indicated that reduction of LPS and restoration of intestinal TJP may be a novel therapeutic strategy for treatment of liver fibrosis development in NASH.

## Introduction

Non-alcoholic fatty liver disease (NAFLD) is the most common type of liver disease in the general population (1). NAFLD includes simple steatosis, non-alcoholic steatohepatitis (NASH), cirrhosis and hepatocellular carcinoma (HCC) (2). Although NAFLD is benign, it has been reported that 20% of patients with NAFLD progress to NASH, cirrhosis and HCC (3,4). The pathophysiological events and effective therapies for NASH remain to be elucidated.

Previous clinical studies have reported that endotoxin/toll-like receptor 4 (TLR4) signaling is crucial in the activation of inflammatory pathways associated with NASH (5). TLR4 is a pattern recognition receptor, which recognizes endotoxin and signals through adaptor molecules, termed myeloid differentiation primary response gene 88 and Toll/interleukin-1 receptor domain-containing adaptor-inducing interferon-β, to activate transcription factors that initiate innate immunity (6). TLR4 is expressed in multiple liver cell types, including liver vascular endothelial cells, Kupffer cells and hepatic stellate cells (HSC) (7,8). The effect of TLR4 on HSC is integral to fibrosis development through its effects on transforming growth factor-β (TGF-β)-dependent collagen production (8).

In human and animal studies, it has been reported that NASH is associated with portal LPS levels through mechanisms involving bacterial translocation (9,10) and the gut microbiota is considered to generate products such as lipopolysaccharide (LPS), a cell-wall component of Gram-negative bacteria, which is delivered into the liver via the portal vein (11,12). Endotoxin production by gut microbiota may cause inflammation in patients with obesity, diabetes, metabolic disorder, NAFLD and NASH (11,13). Plasma LPS levels are associated with small intestinal bacteria overgrowth, the change of composition of the microbiota and increased intestinal permeability (14).

Polymyxins are antibiotics with a structure consisting of a cyclic peptide with a long hydrophobic tail. They are selectively toxic to Gram-negative bacteria, including *Escherichia coli, Pseudomonas aeruginosa*, *Klebsiella pneumoniae* and other members of the Enterobacteriaceae family due to their specificity for the LPS molecule, which exists within a number of Gram-negative outer membranes. They are produced by nonribosomal peptide synthetase systems in Gram-positive bacteria, including *Paenibacillus polymyxa* and disrupt the structure of the bacterial cell membrane by interacting with phospholipids. They are not absorbed through the gastrointestinal tract. In clinical settings, they are used for patients with Gram-negative bacterial infections and for endotoxin apheresis column treatment of endotoxemia (15).

Neomycins are aminoglycoside antibiotics and are effective against Gram-negative and Gram-positive bacteria. They are produced by Gram-positive bacteria, including *Streptomyces fradiae*. They inhibit the protein synthesis of bacteria via binding to 30S ribosomes. Their absorption through the gastrointestinal tract is limited and they are useful for Gram-negative bacterial infections in clinical settings.

In the present study, the effects of these poorly absorbed antibiotics on intestinal permeability and on the progression of liver fibrosis were assessed. The results revealed that, in a rat model of choline deficient amino acid-induced liver fibrosis, the administration of poorly absorbed antibiotics led to reduced intestinal permeability and decreased liver fibrosis. Consequently, the present study elucidated the role of LPS in the pathogenesis of NASH.

## Materials and methods

### Animal model of liver disease

Male six-week-old Fischer 344 rats (CLEA Japan, Inc., Osaka, Japan) were housed in a room under controlled temperature and a 12/12 h light-dark cycle. The animals were divided into the following three experimental groups and fed for 8 weeks: i) Choline-deficient amino acid diet (CDAA; n=10); ii) choline-deficient amino acid diet plus antibiotics (CDAA+AB; n=10) and iii) choline-supplemented amino acid diet (CSAA; n=5). All rats were sacrificed at the end of week 8. For selective intestinal decontamination, poorly absorbable antibiotics (1 g/l polymyxin B sulfate salt (Fluka, Buchs, Switzerland) and 3 g/l neomycin trisulfate salt hydrate (Sigma-Aldrich, St. Louis, MO, USA) were administered to the rats in the CDAA+AB group by adding them to drinking water during the experimental period, excluding the first and fifth week. All animal procedures were performed according to standard protocols and in accordance with the standard recommendations for the proper care and use of laboratory animals. This study was approved by the ethics committee of Nara Medical University, Kashihara, Japan.

### Histological examination

Conventional histological examination was performed using hematoxylin and eosin and Sirius-red (Narabyouri Research, Nara, Japan) staining of the excised liver sections, as described previously (16).

### Immunohistochemistry

For immunostaining of the α-smooth muscle actin (α-SMA), 5 μm-thick liver sections were stained using the indirect immunoperoxidase technique with mouse anti-human monoclonal anti-α-SMA antibody (undiluted; Dako Japan, Co., Ltd., Kyoto, Japan), as described previously (16). For the immunofluorescence examination, frozen liver and intestinal sections were fixed with 4% paraformaldehyde for 10 min at 4°C and blocked with 3% bovine serum albumin for 1 h at room temperature to eliminate background staining. The tissue sections were then incubated with primary antibodies rabbit anti-rat polyclonal zona occludens antibody (ZO-1; 1:100; Invitrogen Life Technologies, Carlsbad, CA, USA) and mouse anti-rat monoclonal Claudin-4 antibody (1:100; Invitrogen Life Technologies) at 4°C overnight. This was followed by incubation with the appropriate donkey anti-rabbit Alexa Fluor-488 or goat anti-mouse Alexa Fluor-546 secondary antibodies (1:200; Invitrogen Life Technologies) for 1 h at room temperature. The nuclei were counterstained with 4′,6-diamidino-2-phenylindole Fluoromount-G (Southern Biotech, Birmingham, AL, USA). The immunofluorescent staining was visualized using a Zeiss Axiovert 40 CEL^®^ microscope (Zeiss, Jena, Germany) and images from ZO-1 and Claudin-4 staining were quantified using Axio software version 4^®^ (Zeiss). For quantification, five images were randomly selected for quantification analysis from each sample and the software program quantified the staining intensity of the selected images based on a preselected threshold.

### Reverse transcription quantitative polymerase chain reaction (RT-qPCR)

Total RNA was extracted from the liver and intestinal tissue samples using acid guanidinium thiocyanate-phenol-chloroform extraction. The mRNA levels of collagen Iα, TGF-β, TLR4 and LPS-binding protein (LBP) in the liver and TLR4 in the intestine were measured by qPCR using the Applied Biosystems StepOnePlus™ Real-Time PCR^®^ (Applied Biosystems, Foster City, CA, USA), as described previously (17). Primer sequences were as follows: β-actin, forward 5′-GGA GAT TAC TGC CCT GGC TCC TA-3′ and reverse 5′-GAC TCA TCG TAC TCC TGC TTG CTG-3′; TLR4, forward 5′-CCG CTC TGG CAT CAT CTT CA-3′ and reverse 5′-CCC ACT CGA GGT AGG TGT TTC TG-3′; LBP, forward 5′-AAC ATC CGG CTG AAC ACC AAG-3′ and reverse 5′-CAA GGA CAG ATT CCC AGG ACT GA-3′; TGF-β, forward 5′-CGG CAG CTG TAC ATT GAC TT-3′ and reverse 5′-AGC GCA CGA TCA TGT TGG AC-3′ and collagen Iα, forward 5′-AGC TCC TGG GCC TAT CTG ATG A-3′ and reverse 5′-AAT GGT GCT CTG AAA CCC TGA TG-3′. The cycling conditions were as follows: Initial holding stage at 95°C for 20 sec; followed by 40 cycles of 95°C for 3 sec and 60°C for 30 sec; followed by the melting curve stage of 95°C for 15 sec, 60°C for 1 min and 95°C for 15 sec.

### Protein expression analysis

The hepatic tissue was homogenized in lysis buffer (Tissue Protein Extraction Reagent; Thermo Scientific, Kanagawa, Japan) containing a mixture of protease and phosphatase inhibitors (Roche Diagnostics, Basel, Switzerland). The total collagen volume in the liver was measured using a Sircol collagen assay kit^®^ (Biocolor Ltd., Carrickfergus, Northern Ireland). The TGF-β levels in the liver were measured using ELISA (R&D Systems, Minneapolis, MN, USA).

### Determination of rat intestinal permeability

A total of 25 mg fluorescein isothiocyanate (FITC)-dextran (40 kDa; Sigma-Aldrich) was orally administered per animal on the day of sacrifice. At 4 h after oral gavage of FITC-dextran, each rat was anesthetized and blood was drawn from its portal vein. The plasma was analyzed by fluorescence measurement at an excitation wavelength of 490 nm and an emission wavelength of 520 nm.

### Statistical analysis

The results are presented as the mean ± standard deviation and were analyzed using Student’s t-test for unpaired data (SPSS version 22; IBM, Armonk, NY, USA). P<0.05 was considered to indicate a statistically significant difference.

## Results

### General findings

The general findings of each experimental group at the time of sacrifice are shown in Table I. The relative weights of the liver in the CDAA group and the CDAA+AB group were significantly higher than that of the CSAA group, whereas no significant differences were observed between the CDAA and the CDAA+AB groups. Regarding the serological data between the CDAA group and the CDAA+AB group, no significant differences were observed in the levels of aspartate aminotransferase, alanine aminotransferase, albumin, total bilirubin, glucose, triglyceride, total cholesterol or high-density lipoprotein cholesterol.

### Effect of poorly absorbable antibiotics on liver fibrosis development

**Figure 1:**
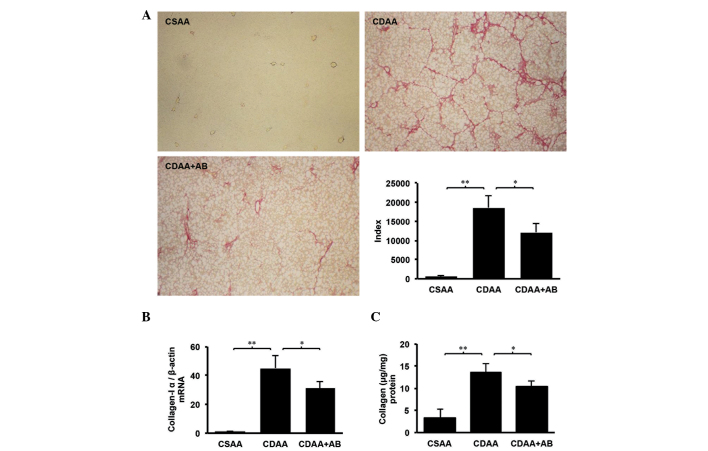
ABs ameliorate liver fibrosis induced by a CDAA diet. (A) Collagen deposition was evaluated by Sirius-red staining. Extensive fibrosis was observed in the CDAA group. Treatment with ABs revealed a significant inhibitory effect against liver fibrosis. No fibrosis was observed in the CSAA group. Semi-quantitative analysis confirmed histological findings. (B) Collagen-Iα mRNA expression in the liver was significantly increased in the CDAA group when compared with the CSAA group. Treatment with ABs markedly suppressed the expression of collagen-Iα. (C) Compared with the CSAA group, the total collagen level of the CDAA group was increased and this increase was significantly suppressed by ABs. Data are presented as the mean ± standard deviation. ^*^P<0.05 vs. CDAA, ^**^P<0.01 vs. CSAA. CDAA, choline deficient amino acid; CSAA, choline supplemented amino acid; AB, antibiotic.

**Figure 2:**
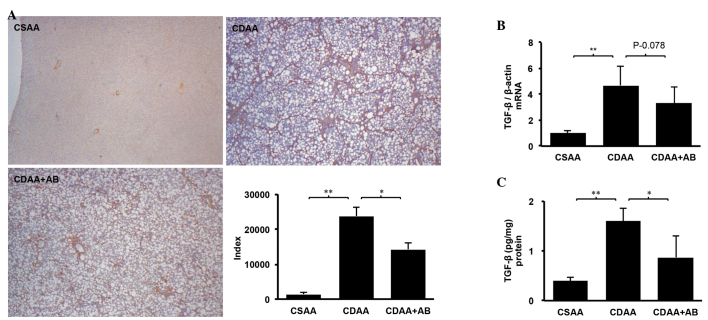
Activated hepatic stellate cells are reduced following administration of ABs. (A) Compared with the CDAA group, a significantly decreased number of α-SMA immunopositive cells were observed following AB treatment. No α-SMA immunopositive cells were observed in the CSAA group. Semi-quantitative analysis confirmed that α-SMA immunopositive cells were decreased in the antibiotic-treated group in parallel with the reduction of liver fibrosis. (B) Compared with the CSAA group, significantly increased TGF-β mRNA expression in the liver of the CDAA group was demonstrated. Treatment with antibiotics suppressed the expression of TGF-β compared with the CDAA group. (C) Increased TGF-β protein level in the CDAA group was significantly suppressed by ABs. The degree of TGF-β suppression by ABs was at a similar magnitude of the inhibition of α-SMA positive cells. Data are presented as the mean ± standard deviation. ^*^P<0.05 vs. CDAA, ^**^P<0.01 vs. CSAA. CDAA, choline deficient amino acid; CSAA, choline supplemented amino acid; AB, antibiotic; TGF, transforming growth factor; α-SMA, α-smooth muscle actin.

The present study initially examined the effects of poorly absorbable antibiotics on liver fibrosis, induced by CDAA intake. As shown in Fig. 1A, although marked fibrosis was observed in the CDAA group, no fibrosis was identified in the CSAA control group and poorly absorbable antibiotics attenuated the CDAA-induced fibrosis. Since it is generally accepted that activated HSCs are critical in fibrogenesis, immunohistochemical analysis of α-SMA was performed to examine the effects of poorly absorbable antibiotics on HSC activation during the development of liver fibrosis. Markedly decreased levels of α-SMA expression were observed in the CDAA+AB group (Fig. 2). Semi-quantitative analysis performed using Image J software version 64 (National Institutes of Health, Bethesda, MD, USA) revealed a significant decrease of α-SMA in the CDAA+AB group compared with the CDAA group (Fig. 2A). Additionally, markedly suppressed levels of hepatic TGF-β and total collagen were revealed in the CDAA+AB group, compared with the CDAA group (Figs. 1C and 2C). RT-qPCR also revealed that these inhibitory effects were closely correlated with alterations in mRNA expression levels of TGF-β and collagen-Iα (Figs. 1B and 2B). The results of the present study suggested that poorly absorbable antibiotics attenuated HSC activation and liver fibrosis via control of TGF-β and collagen in the experimental hepatic fibrosis model.

### Effect of poorly absorbable antibiotics on LPS-TLR4 signaling

**Figure 3:**
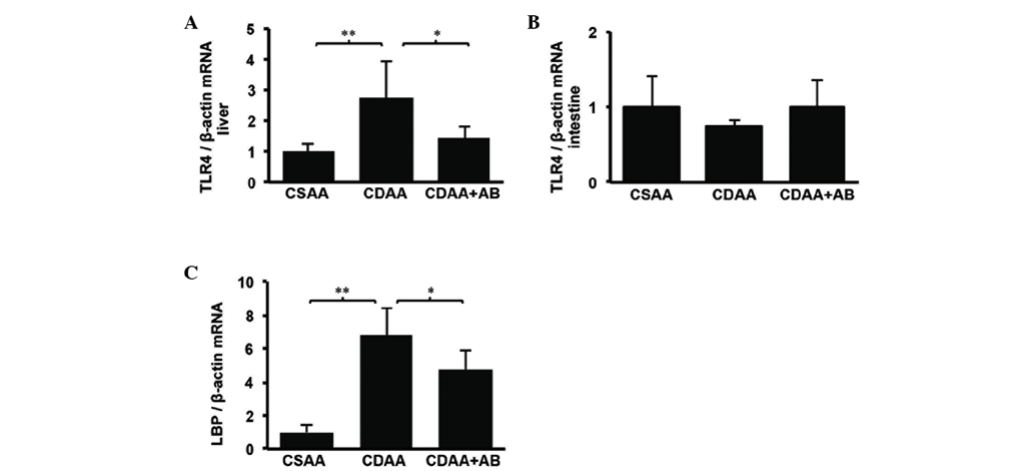
ABs attenuate LPS-TLR4 signaling. (A) TLR4 mRNA expression in the liver of the CDAA group was significantly increased when compared with the CSAA group. Treatment with ABs suppressed the expression of TLR4 in the liver. However, (B) TLR4 mRNA level in the small intestine was not different among all groups. (C) LBP mRNA level was elevated in the CDAA group when compared with the CSAA group and this increase was significantly decreased by ABs. Data are presented as the mean ± standard deviation. ^*^P<0.05 vs. CDAA, ^**^P<0.01 vs. CSAA. CDAA, choline deficient amino acid; CSAA, choline supplemented amino acid; LPS, lipopolysaccaride; TLR4, toll-like receptor 4; LPB, LPS-binding protein.

TLR4 enhances hepatic inflammation and fibrogenesis (8,18). This finding led to the hypothesis of the present study that poorly absorbable antibiotics may attenuate LPS-TLR4 signaling, with liver fibrosis ameliorated as a result. TLR4 mRNA expression in the liver and intestine were then examined. Notably, TLR4 mRNA level in the liver was elevated in the CDAA group and the CDAA-induced increase was significantly decreased by antibiotics (Fig. 3A). However, TLR4 mRNA levels in the intestine were similar in all groups (Fig. 3B). These data suggested that TLR4-associated signaling in the intestine was not important for liver fibrosis, whereas TLR4 in the liver was essential for liver fibrosis. Subsequently, the mRNA levels of LBP were measured, which is essential for LPS to bind TLR4 and is correlated with serum endotoxin levels (24). A significantly elevated mRNA level of LBP was observed in the CDAA group and this increase was reduced in the CDAA+AB group (Fig. 3C).

### Effect of poorly absorbable antibiotics on intestinal permeability and tight junction protein (TJP)

**Figure 4:**
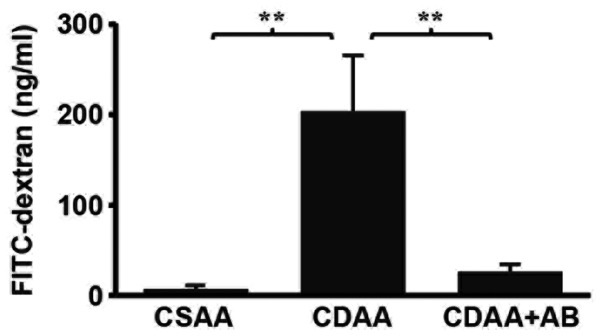
ABs improve intestinal permeability. The portal fluorescent level in the CDAA group was significantly increased compared with the CSAA group. The increase of intestinal permeability in the CDAA group was improved by poorly absorbable ABs. Data are presented as the mean ± standard deviation. ^**^P<0.01. CDAA, choline deficient amino acid; CSAA, choline supplemented amino acid; AB, antibiotic; FITC, fluorescein isothiocyanate.

**Figure 5:**
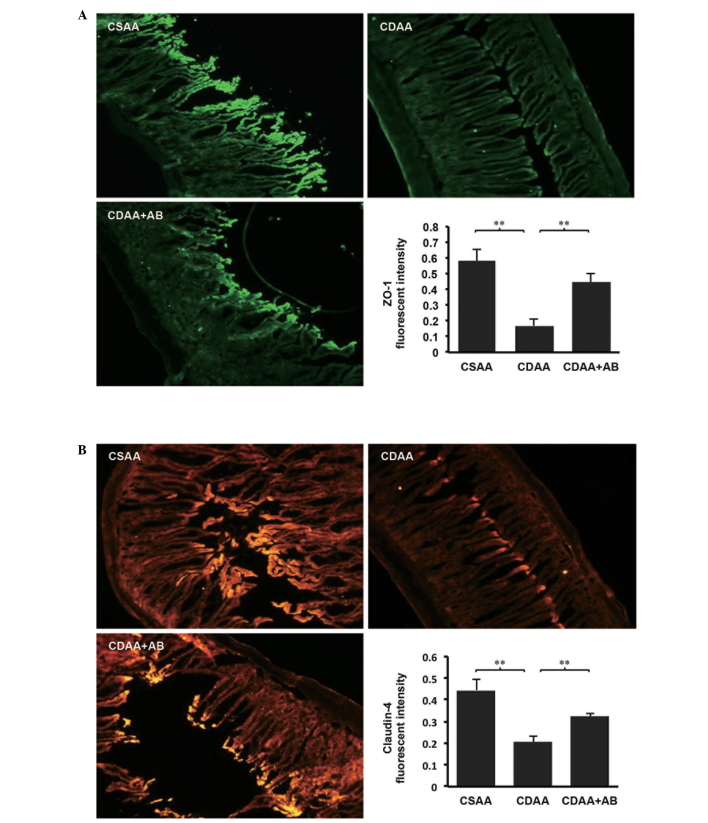
ABs improve tight junction protein expression in the small intestine. (A and B) Immunohistochemical analyses demonstrated adequate expression of ZO-1 and Claudin-4 in the intestinal sections of the CSAA control group. ABs markedly improved the delocalization and substantially decreased the intestinal sections of the CDAA rats. Semi-quantitative analysis confirmed immunohistochemical findings. Data are presented as the mean ± standard deviation. ^**^P<0.01. CDAA, choline deficient amino acid; CSAA, choline supplemented amino acid; AB, antibiotic; ZO-1, zona-occludens 1.

The elevated mRNA level of LBP suggested that the serum LPS level was increased in the CDAA group. The serum LPS level was hypothesized to be involved in gut permeability, therefore gut permeability was examined by analyzing the fluorescence levels of the portal vein following oral gavage loading with FITC-dextran. The fluorescence levels of the portal vein in the CDAA group were increased when compared with the CSAA group. The increase of intestinal permeability of the CDAA group was improved by the addition of poorly absorbable antibiotics (Fig. 4). Since gut permeability is controlled by TJPs, including ZO-1 and Claudin-4 (14,20), the immunohistochemical analyses of ZO-1 and Claudin-4 in the intestinal sections were examined. As shown in Fig. 5, immunohistochemical analyses revealed that significant expression levels of ZO-1 and Claudin-4 were predominant in the intestinal sections of the CSAA control group (Fig. 5A and B). By contrast, the delocalization and substantial decrease in the intestinal sections of the CDAA group were significantly improved by poorly absorbable antibiotic administration.

## Discussion

In the present study, the effect of the poorly absorbable antibiotics, polymyxin and neomycin, on the development of hepatic fibrosis and intestinal permeability was examined. It was demonstrated that the antibiotics not only reduced CDAA-induced hepatic fibrosis and HSC activation but also improved intestinal permeability.

The liver is the main target of intestinally-derived bacterial products and the rate of bacterial translocation increases in various models of hepatic disease, rendering LPS a possible candidate mediator of TLR4-dependent profibrogenic effects. Accordingly, increased LBP mRNA expression was identified in the CDAA group, indicating that LPS was increased. In addition, LBP mRNA expression and fibrogenesis were reduced in rats treated with poorly absorbable antibiotics, suggesting that the intestinal flora is the main source of LPS and that intestinally-derived LPS drives fibrogenesis.

Translocated LPS derived from the gut microflora is considered to mediate TLR4 activation in the liver. However, this translocation may be independent of intestinal TLR4 (21). The mRNA expression levels of TLR4 in the liver and intestine were assessed in the present study. The mRNA expression levels in the liver of the CDAA-induced NASH model were increased. By contrast, the mRNA levels in the intestine of the CDAA-induced NASH model were not increased. However, Guo *et al* (22) reported that LPS caused an increase in intestinal permeability via an intracellular mechanism involving the TLR4-dependent upregulation of CD14 membrane expression. The association between LPS and TLR4 in intestinal permeability remains controversial.

NAFLD is associated with increased intestinal permeability and small intestinal bacteria overgrowth (21,23). These findings have been considered to be associated with the severity of hepatic steatosis. Increased intestinal permeability may be a condition supporting the hypothesis of the contribution of the gut-liver axis to the development of NAFLD (14). The intestinal barrier defect may be caused by disruption of the tight junction proteins between intestinal epithelial cells, allowing substances, including lipopolysaccharides, to pass from the intestine to the portal vein, imbalance of proliferation and apoptosis, intestinal mucosal atrophy and edema, which is associated with portal hypertension or the absence of bile acids and systemic increases in inflammatory cytokines and oxidative stress produced in the liver (24–26). LPS causes an increase in intestinal permeability via an intracellular mechanism involving the TLR4-dependent upregulation of CD14 membrane expression (22).

Caco-2 cells grown in zinc-deficient media have reduced transepithelial electrical resistance and altered expression levels of ZO-1 and occludin, which are intestinal tight junction proteins, compared with Caco-2 cells grown in zinc-replete media (27). In clinical practice, zinc deficiency is common in patients with liver cirrhosis (28,29). In *in vitro* study, Caco-2 cells, which mimic intestinal epithelial cells, grown in zinc-deficient media have reduced TJP; therefore it was hypothesized that zinc deficiency may potentially be relevant to the increased intestinal permeability. In the NASH model used in the present study, CDAA-induced hepatic fibrosis, endogenous LPS and systemic increases in inflammatory cytokines may disrupt intestinal tight junction proteins. Considering these findings, the recruitment of TJPs using probiotics and zinc preparations, for example, offers a novel strategy for NASH treatment.

The intestinal microflora is involved in liver fibrosis. In the present *in vivo* model, dietary habits, which increase the percentage of intestinal endotoxin producers, including Gram-negative bacteria may accelerate liver fibrogenesis, introducing dysbiosis as a cofactor contributing to chronic liver injury in NAFLD (30). Endo *et al* (9) also demonstrated that butyrate-producing probiotics reduced NAFLD progression in rats. These data indicated that intestinal microflora may be a new target for NASH treatment.

In conclusion, the inhibition of LPS-TLR4 signaling with poorly absorbable antibiotics attenuated the liver fibrosis development in NASH via inhibition of HSC activation. These results indicated that reduction of LPS and restoration of the intestinal TJPs may be a new therapeutic strategy for the treatment of the development of liver fibrosis in NASH.

## Figures and Tables

**Table I:** Characteristic features of the experimental groups.

Characteristics	CSAA (n=5)	CDAA (n=10)	CDAA+AB (n=10)
Body weight (g)	304.0±11.6	291.3±18.5	250.6±12.4^a^
Liver weight (g)	10.4±0.7	18.6±1.4^a^	15.6±1.5^a^
Liver weight (% body)	3.4±0.2	6.4±0.2^a^	6.2±0.4^a^
Aspartate aminotransferase (IU/l)	57.6±6.0	361.2±39.0^a^	384.5±46.3^a^
Alanine aminotransferase (IU/l)	25.4±8.6	244.8±55.2^a^	259.4±53.0^a^
Total bilirubin (mg/dl)	0.03±0.01	0.13±0.02^a^	0.13±0.01^a^
Albumin (g/dl)	3.0±0.2	3.3±0.3	3.1±0.2
Total C (mg/dl)	42.6±5.9	26.1±3.3^a^	24.6±2.1^a^
High density lipoprotein C (mg/dl)	13.4±3.2	14.3±2.5	14.9±1.6
Triglyceride (mg/dl)	116.6±18.9	10.6±7.6^a^	6.4±1.6^a^
Glucose (mg/dl)	135.0±28.8	101.9±11.3	105.3±29.6

Data are presented as the mean ± standard deviation.

a P<0.01, compared with CSAA.

C, cholesterol; CDAA, choline deficient amino acid; CSAA, choline supplemented amino acid; AB, antibiotics.

## Abbreviations

TLR4: toll-like receptor 4
NASH: non-alcoholic steatohepatitis
LPS: lipopolysaccharide
CDAA: choline deficiency amino acid
HSC: hepatic stellate cell
LBP: LPS binding protein
CSAA: choline supplemented amino acid
TJP: tight junction protein
NAFLD: non-alcoholic fatty liver disease
HCC: hepatocellular carcinoma
TGF-β: transforming growth factor-β
α-SMA: α-smooth muscle actin
Glu: glucose
TG: triglyceridel
